# Antifeedant Effects of Essential Oil, Extracts, and Isolated Sesquiterpenes from *Pilgerodendron uviferum* (D. Don) Florin Heartwood on Red Clover Borer *Hylastinus obscurus* (Coleoptera: Curculionidae)

**DOI:** 10.3390/molecules23061282

**Published:** 2018-05-27

**Authors:** Javier Espinoza, Alejandro Urzúa, Leonardo Bardehle, Andrés Quiroz, Javier Echeverría, Marcia González-Teuber

**Affiliations:** 1 Laboratorio de Ecología Química, Departamento de Ciencias Químicas y Recursos Naturales, Universidad de La Frontera, Casilla 54-D, Temuco 4811230, Chile; leonardo.bardehle@ufrontera.cl (L.B.); andres.quiroz@ufrontera.cl (A.Q.); 2 Centro de Excelencia en Investigación Biotecnológica Aplicada al Medio Ambiente (CIBAMA), Universidad de La Frontera, Casilla 54-D, Temuco 4811230, Chile; 3 Laboratorio de Química Ecológica, Departamento de Ciencias del Ambiente, Universidad de Santiago de Chile, Casilla 40, Correo 33, Santiago 9170022, Chile; javier.echeverriam@usach.cl (J.E.); marcia.gonzalez.t@usach.cl (M.G.-T.); 4 Scientific and Technological Bioresource Nucleus, BIOREN-UFRO, Universidad de La Frontera, Temuco 4811230, Chile; 5 Instituto de Investigación Multidisciplinar en Ciencia y Tecnología, Universidad de La Serena, La Serena 1720256, Chile

**Keywords:** *Hylastinus obscurus*, red clover, essential oils, sesquiterpenes, antifeedant effects, lipophilicity, vapor pressure

## Abstract

The beetle *Hylastinus obscurus* Marsham (Coleoptera: Curculionidae), endemic to Europe and Northern Africa, is one of the most important red clover pests in Chile. As commercial insecticides are less effective against this pest, plant secondary metabolites have been considered as an alternative for its control. Here, we have investigated the chemical composition of essential oil (EO), petroleum ether extract (PEE), and dichloromethane extract (DCME) from *Pilgerodendron uviferum* heartwood. Additionally, the effects of EO and extracts on the feeding behavior (% of weight shift) of *H. obscurus* have been evaluated. The composition of EO, PEE, and DCME were analyzed using gas chromatography (GC) and gas chromatography/mass spectrometry (GC/MS). The results showed the presence of a similar mixture of sesquiterpenes in the essential oil and in both of the extracts, which accounted for circa 60% of the total mixture. Sesquiterpenes were further isolated using chromatographic methods and were structurally characterized by optical rotation, GC–MS, FTIR, and 1D and 2D NMR experiments. The physicochemical properties of the isolated sesquiterpenes, including lipophilicity and vapor pressure, were also determined. The sesquiterpenes were identified as the following: (−)-*trans*-calamenene (**1**), cadalene (**2**), (−)-cubenol (**3**), (−)-*epi*-cubenol (**4**), (−)-torreyol (**5**), and (−)-15-copaenol (**6**). The antifeedant activity of EO, extracts, and isolated sesquiterpenes were evaluated using artificial diets in a non-choice test. Relative to the control, the EO, DCME extract, and the isolated sesquiterpenes, namely, (−)-*trans*-calamenene (**1**), cadalene (**2**), and (**5**) torreyol, were found to be the most effective treatments against *H. obscurus*. Our study showed that the compounds occurring in *P. uviferum* heartwood were effective in reducing the adult growth of *H. obscurus*. The physicochemical properties of the isolated sesquiterpenes might have been associated with antifeedant effects.

## 1. Introduction

*Trifolium pratense L.* (Fabaceae), commonly known as red clover, is an important perennial forage crop, which is cultivated for its nitrogen fixation, high forage quality, and beneficial effects on soil structure [1]. It is endemic to Western Asia, Northwest Africa, and Southeast Europe [2]. It is mostly cultivated in warm areas of the world, either as a monoculture or as a mixture with other forage crops [3]. Despite being a perennial plant, this crop does not exceed more than three years of survival [3]. An infestation by *Hylastinus obscurus* Marsham (Coleoptera: Curculionidae), or red clover borer, is considered as one of the most important factors that is contributing to the decay of red clover [3,4], and it is widely considered to be the most serious pest of this crop globally [5]. In the last decade, there has been increased interest in the use of alternatives as opposed to conventional chemical insecticides, which are considered environmentally deleterious. Plant-derived substances, which are capable of repelling and/or killing insect pests, have been shown, in several studies, to be effective substitutes for chemical pesticides [6,7,8,9,10]. 

In Chile, *H. obscurus* causes a premature decay of *T. pratense*, which leads to a significant yield reduction [11,12]. Both the larval and adult stages feed on roots, which causes weakening and subsequent plant mortality [13]. Furthermore, red clover pastures can suffer infestation levels of approximately 70–100%, after a period of 2–3 years [13]. To date, the chemical control of *H. obscurus* has largely been unsuccessful [14] and biological control has hardly been explored [15]. However, some studies have shown that the secondary metabolites that are present in red clover roots play an important role in *H. obscurus* behaviour. For example, some isoflavones, depending on their structure, elicited an antifeedant effect, while others elicited phagostimulant insect behavior. The same pattern has been observed in the volatile fraction that was obtained from the *T. pretense* roots [14]; where some volatiles have acted as repellents against *H. obscurus*, others have stimulated its activity. Alternative strategies to effectively control the feeding behavior in *H. obscurus* have so far hardly been explored, and they are a high priority for red clover producers.

The aim of this study was to investigate the potential antifeedant effects of plant secondary metabolites on *H. obscurus*. We studied the effects of essential oil (EO), petroleum ether extract (PEE), dichloromethane extract (DCME), and six purified sesquiterpenes from the heartwood of *Pilgerodendron uviferum* (D. Don) Florin (Cupressaceae), on the feeding behavior of the red clover borer. *P. uviferum*, a conifer native to Southern Chile and Argentina, which is known to be highly resistant to microorganisms and insects [16,17]. Therefore, this tree may be a potential source of natural products for the control of the red clover borer. In fact, a recent study has shown a repellent effect for the essential oil of *P. uviferum* against the weevil *Aegorhinus superciliosus* [18], a major berry pest in Chile. The composition and characterization of EO and extracts of *P. uviferum* heartwood were determined using gas chromatography (GC) and gas chromatography/mass spectrometry (GC/MS). The structural identification of the sesquiterpenes were determined using optical rotation, FTIR, and 1D and 2D NMR experiments. Some of the physicochemical properties of the sesquiterpenes, including lipophilicity and vapor pressure (logVP), were also determined. The antifeedant activities of the EO, extracts, and pure sesquiterpenes against the antifeeding behavior of the *H. obscurus* adults were performed using a no-choice test that was based on artificial diets.

## 2. Results

### 2.1. Chemical Analyses

**Figure 1:**
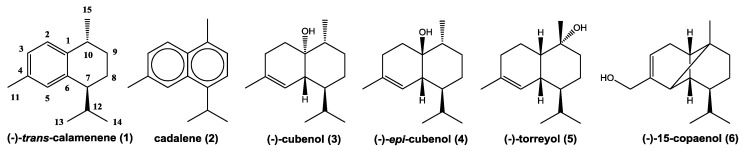
Sesquiterpenes isolated from heartwood of *Pilgerodendron uviferum*.

The EO composition of *P. uviferum* was obtained from Espinoza et al. [18]. According to these analyses, three monoterpenes (0.43%) and 17 sesquiterpenes (86.62%) were present in EO, with torreyol (24.16%), cubenol (22.64%), 15-copaenol (15.46%), and δ-cadinene (10.81%) being the most abundant components [18]. In this study, we identified 25 and 22 compounds in the PEE and DCME extracts, respectively (Table 1), accounting for 82.4% and 79.8% of all of the detected compounds, respectively. There were 19 sesquiterpenes (73.53%, comprising 13 non-oxygenated and 6 oxygenated) and 6 diterpenes (8.90%, comprising one non-oxygenated and 5 oxygenated) that were present in the PEE extract, and 17 sesquiterpenes (28.57%, from which 11 non-oxygenated and 6 oxygenated) and 5 oxygenated diterpenes (47.97%) that were present in the DCME extract. Sesquiterpenes δ-cadinol, cubenol, and 15-copaenol were the most abundant compounds in the PEE and DCME extracts (Table 1, Figure S1). The most abundant sesquiterpenes that were isolated from the EO and both of the extracts of *P. uviferum* heartwood were further characterized using optical rotation, MS, FTIR, monodimensional ^1^H, ^13^C-NMR spectrums and DEPT 135, COSY, and bidimensional NMR spectrum analyses, including HSQC-ed and HMBC (Table 2 and Table 3). Pure sesquiterpenes that were isolated from heartwood comprised (−)-*trans*-calamenene (**1**), cadalene (**2**), (−)-cubenol (**3**), (−)-*epi*-cubenol (**4**), (−)-torreyol (**5**), and (−)-15-copaenol (**6**) (Figure 1). The structural assignments were corroborated by the information that was published in the literature, as follows: (−)-*trans*-calamenene (**1**) [19,20,21], cadalene (**2**) [22], (−)-cubenol (**3**) and (−)-*epi*-cubenol (**4**) [16,19,23,24], (−)-torreyol (**5**) [16,19,25], and (−)-15-copaenol (**6**) [16,19].

### 2.2. Antifeedant Bioassays

**Figure 2:**
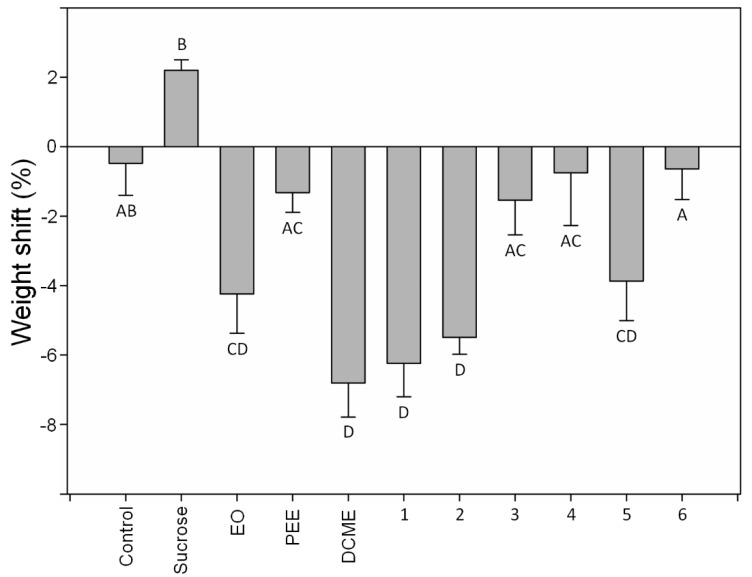
Weight shift (%) of *H. obscurus* adults fed with artificial diet supplemented with 10 µL of EO, PEE, DCME, and 1–6 sesquiterpenes that were diluted in CH_2_Cl_2_ (1000 mg/L). Control: artificial diet supplemented with 10 µL of CH_2_Cl_2_. Sucrose: artificial diet supplemented with 10 µL of sucrose diluted in H_2_0 (1000 mg/L). The sesquiterpenes were (−)-*trans*-calamenene (**1**), cadalene (**2**), (−)-cubenol (**3**), (−)-*epi*-cubenol (**4**), (−)-torreyol (**5**), and (−)-15-copaenol (**6**). Values indicate mean + SE. Different letters indicate significant differences based on the Tukey HSD test.

**Figure 3:**
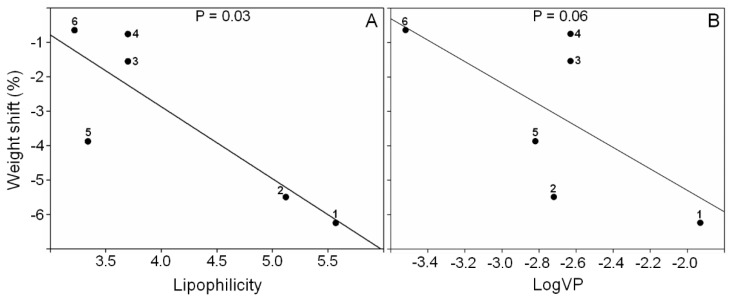
(**A**) Correlations between weight shift (%) and lipophilicity and (**B**) between weight shift (%) and LogVP. For logVP, values near to zero indicated higher vapor pressure. The sesquiterpenes were (−)-*trans*-calamenene (**1**), cadalene (**2**), (−)-cubenol (**3**), (−)-*epi*-cubenol (**4**), (−)-torreyol (**5**) and (−)-15-copaenol (**6**).

Antifeeding behavior of the *H. obscurus* adults was significantly affected by diet (F_1.86_ = 9.52, *p* = 0.000, one-way ANOVA). In comparison to the control, only the essential oil, the DCME extract, and the sesquiterpenes (−)-*trans*-calamenene (**1**), cadalene (**2**), and (−)-torreyol (*5*), had caused a significant reduction in the weight of the *H. obscurus* adults (Figure 2). All of the other treatments were not significantly different from the control. As expected, the sucrose significantly increased the weight of the insect. Regarding the analyzed physicochemical properties, our results revealed that the sesquiterpenes with the highest values of lipophilicity and logVP, including (−)-*trans*-calamenene (**1**) and cadalene (**2**), significantly decreased the weight in *H. obscurus*. Whereas, the feeding behavior of *H. obscurus* (weight shift) was negatively and significantly correlated with lipophilicity (r = −0.84, *p* = 0.03; Figure 3A), where a marginally significant (r = −0.73, *p* = 0.06; Figure 3B) negative tendency between the feeding behavior and logVP were observed.

## 3. Discussion

The main goal of this study was to determine the potential antifeedant effects of the EO and extracts of *P. uviferum* heartwood on the global pest *H. obscurus*. Here we have showed evidence that two of the most abundant sesquiterpenes that were found in EO and both of the extracts were effective in controlling the feeding behavior of the red clover borer. As in other species of Cupressaceae that were characterized by terpenoid biosynthesis, the composition of the PEE and DCME extracts from *P. uviferum* heartwood demonstrated high levels of sesquiterpenes. This agreed with chemical analyses of *P. uviferum* heartwood that was described in a previous study [18], which reported the presence of ten sesquiterpenes and five diterpenes in a heartwood methanol extract. In addition to this, our study identified eleven sesquiterpenes that were previously undescribed in this species.

The weight loss that was produced by the EO, the non-polar extract DCME, and sesquiterpenes *trans*-calamenene (**1**) and cadalene (**2**), was not uncommon. Several studies had demonstrated that the essential oils were an effective inhibitor of the feeding behavior in insects. For example, the antifeedant effects of a variety of plant EOs were reported in the weevils *Sitophilus granarius* and *S. zeamais* (Coleoptera), which were common pests of stored grain [27,28,29]. Furthermore, Shukla et al. [30] demonstrated that the EOs from the aerial parts of *Eupatorium adenophorum* and *Artemisia nilagirica*, which were abundant in monoterpenes and sesquiterpenes, produced an antifeedant effect in *Rhynchophorus ferrugineus*, which was a major pest of coconut palm. A recent study revealed that several sesquiterpenes that were commonly found in aromatic Mediterranean plants, induced an antifeedant activity in the Colorado potato beetle *Leptinotarsa decemlineata* [31], with the sesquiterpene (−)-α-bisabolol, which was found to be the most active compound against this beetle, followed by the monoterpenoids carvacrol, (+)-terpinen-4-ol, and thymol.

*H. obscurus* was one of the most serious red clover pests globally. In Chile, currently, there has been no efficient method for the control of this insect, although semiochemicals appeared to offer a promising alternative for reducing the crop damage that was caused by this curculionid. Toledo et al. [32] showed that long chain fatty acids from non-polar root extracts of *T. pratense* induced feeding behavioral changes (repellent or phagostimulant) in *H. obscurus*, although significant decreases in weight only occurred when palmitic acid was added to artificial diets. Similar results were obtained by Quiroz et al. [15], using the isoflavones that were present in red clover roots, they found that only the isoflavones formononetin and genistein demonstrated inhibiting effects on feeding. Here we showed that the *P. uviferum* EO, DCME extract, and individual sesquiterpenes efficiently deterred the feeding in *H. obscurus*, with (−)-*trans*-calamenene (**1**), cadalene (**2**), and (−)-torreyol (**5**) being the strongest inhibitors in this respect. The behavioral responses to these sesquiterpenes were similar, which resulted in a decrease in insect weight from −0.25% (control) to approximately −5–7%, similar to the levels that were reported for the root isoflavones [15]. The physicochemical properties of these sesquiterpenes, including lipophilicity and vapor pressure, might have been responsible for the antifeedant effects on *H. obscurus*. Our results showed that the negative responses in adults were associated with the sesquiterpenes that had the highest lipophilicity and logVP values. Vapor pressure was a measure of the volatilization tendency of a compound, and might have been useful in estimating the lifespan of the application deposit. The enhanced bioactivity of (−)-*trans*-calamenene (**1**) and cadalene (**2**) seemed to be linked to higher vapor pressure, which led to greater fumigant action and/or lipophilicity, which resulted in better penetration and bioavailability in the insect body. An antifeedant effect of *P. uviferum* EO was previously been reported in the weevil *Aegorhinus superciliosus* (Coleoptera: Curculionidae), a major berry pest in Chile, and in the housefly *Musca domestica* (Diptera: Muscidae) (Urzúa, unpublished results).

Our study showed that the chemical composition of *P. uviferum* heartwood was effective in reducing the adult growth of *H. obscurus*, a major commercial pest of red clover. We suggested that five of our treatments, including EO, DCME extract, (−)-*trans*-calamenene (**1**), cadalene (**2**), and (−)-torreyol (**5**), could have potentially been used in *H. obscurus* control programs in order to prevent or reduce the crop damage in *T. pretense*. Despite the fact that eco-friendly and sustainable alternatives to chemical pesticides have been gaining popularity in Chile, they have remained poorly promoted and need advanced investigation. As a minimum requirement for the development of new biological control agents, future research into the potential of *P. uviferum* compounds in this context should focus on testing the effect of the extracts and sesquiterpenes on both *H. obscurus* and non-target and/or beneficial species under field conditions.

## 4. Material and Methods

### 4.1. Plant and Insect Material

In order to collect adult insects of *H. obscurus*, in November 2014, nine month old *T. pratense* plants were collected from experimental cultivars at the INIA Experimental Station at Carillanca (38°41′ S, 72°25′ W, 200 m.a.s.l.), La Araucanía, Chile. The plants were sampled with enough soil to avoid root damage. Colonies of *H. obscurus* adults, without gender differentiation, were hand collected from the *T. pratense* roots. The beetles were transferred to small cages and were then reared in petri dishes (150 mm diameter × 15 mm length) at 22 °C ± 1 °C under a 16:8 light:dark cycle and 80% humidity. The adults were provided with fresh red clover roots.

### 4.2. Heartwood EO, PEE, and DCME Extraction

The EO that was used here was produced in an earlier study by Espinoza et al. [18]. The light petroleum ether (PEE) and dichloromethane (DCME) extracts were obtained using a Soxhlet extraction, as follows: milled *P. uviferum* heartwood (1.5 kg) was sequentially extracted over a period of 8 h using PEE, followed by DCME; the extracts were dried over anhydrous sodium sulfate, filtered using a frit funnel, and the solvent was evaporated under reduced pressure in a rotatory evaporator, which yielded 42 g of PEE (2.8%) and 25 g of DCME (1.7%).

### 4.3. Sesquiterpene Isolation

The EO and both of the extracts were fractioned by column chromatography with silica gel as a stationary phase, using a different gradient elution for each extract. The EO and PEE gradient elution was as follows: light petroleum ether (PE) (bp 35–60 °C), PE-CH_2_Cl_2_ (1:1), CH_2_Cl_2_, and CH_2_Cl_2_-MeOH (9:1). The DCME gradient elution was as follows: CH_2_Cl_2_ and CH_2_Cl_2_-MeOH step gradient. The resulting fractions were evaporated under reduced pressure and were analyzed using thin layer chromatography on silica gel 60 F_254_ pre-coated plates, using a p-anisaldehyde-sulfuric acid spray reagent for detection. Similar compounds were combined and further purified by column chromatography, with silica gel as a stationary phase to produce (−)-*trans*-calamenene (**1**, 434 mg), cadalene (**2**, 116 mg), (−)-cubenol (**3**, 1.10g), (−)-*epi*-cubenol (**4**, 2.0 g), (−)-torreyol (**5**, 1.2 g), and (−)-15-copaenol (**6**, 2.0 g). The purity of the compounds ranged between 93–97%, according to GC and ^1^H-NMR.

### 4.4. Analyses of Heartwood EO, Extracts, and Sesquiterpenes

Analyses of the EO compounds had previously been performed using gas chromatography (GC) and gas chromatography/mass spectrometry (GC/MS) [18]. For this study, the analyses of the PEE and DCME extracts were performed by GC and GC/MS, using the following instrumentation: a Thermo Electron Model Focus GC (Waltham, MA, USA) coupled to a DSQ Thermo Electron quadrupole mass spectrometric detector, with an integrated data system (Xcalibur 2.0, Thermo Fisher Scientific Inc., Waltham, MA, USA) and a BPX5 capillary column (30 m length, 0.25 µm film thickness, ×0.25 mm i.d., SGE Forte, Trajan Scientific and Medical, Ringwood, Victoria, Australia). The operating conditions were as follows: on-column injection; injector temperature, 250 °C; transfer line temperature, 250 °C; carrier gas, He at 1.00 mL min^−1^; and oven temperature program: 40 °C for 2 min, increased to 250 °C at 5 °C min^−1^, followed by 250 °C for 5 min. The mass spectra were obtained at an ionization voltage of 70 eV. The recording conditions employed a scan time of 1.5 s and a mass range of 30 to 400 amu. The compounds were identified based on the comparisons of the mass spectra with a library database (NIST ver. 2.0, NIST, Gaithersburg, MD, USA), and the comparisons of the calculated retention indices with those that were reported in the literature for the same column type [26].

The sesquiterpenes that were isolated from EO and both of the extracts were analyzed by optical rotation, ATR-FTIR, and ^1^H- and ^13^C-NMR. The specific optical rotation was determined in CH_2_Cl_2_ using a Perkin Elmer 241 spectropolarimeter. The FTIR spectra were recorded on a Perkin Elmer Spectrum 65 spectrometer. Both the ^1^H- and ^13^C-NMR spectra were recorded on a Bruker 400 Ultra Shield spectrometer using CDCl_3_.

### 4.5. Theoretical Estimation of Lipophilicity and Vapor Pressure

The lipophilicity (log P) values were calculated according to the XLOGP3 algorithm using an additive atom mode [33]. The vapor pressure (logVP) was determined using T.E.S.T. [34].

### 4.6. Feeding Bioassays

The feeding bioassays were performed based on a no-choice test using an artificial diet, according to a previously described method [35]. At 24 h prior to the bioassay, thestarved *H. obscurus* adults were placed in petri dishes (100 mm diameter x 15 mm length) on wet filter paper at 5 °C ± 1 °C, in darkness. An artificial diet of 400 μL, which was composed of 87.6% water, 4.3% starch, 3.5% agar, 2.6% glucose, and 2.0% cellulose, was added to a transparent glass tube (6 mm diameter × 25 mm length). Then, 10 μL of EO, PEE, DCME extract, and isolated sesquiterpenes, which were diluted in CH_2_Cl_2_ (1000 mg L^−1^), were added separately to the artificial diet, as proposed by Manosalva et al. [5]. An artificial diet that was supplemented with 10 μL of CH_2_Cl_2_ was used as the control. Sucrose was used as a positive control as it acted as a phagostimulant agent. In this case, 10 μL of sucrose, which was diluted in distillated H_2_O (1000 mg L^−1^), was added to the diet. To ensure a homogeneous distribution of the solution, the glass tubes were vortexed at 12 rpm continuously for 15 sec. After that, the tubes were incubated in the vertical position for 1 h at 21 °C ± 1 °C so as to allow the complete evaporation of the solvent. Subsequently, one previously weighed (iw) *H. obscurus* adult was introduced into each tube, which was then closed with a plastic cap. Each bioassay was replicated 10–15 times, and each insect was used only once. The feeding assay was performed at room temperature over a period of five days under conditions of darkness. Following this, the insects were weighed again (fw). The feeding performance was evaluated by the weight shift (%), as follows:

Weight shift (%) = ((fw − iw)/fw) × 100

### 4.7. Statistical Data Analyses

The differences in the weight shift of *H. obscurus* among different artificial diets were analyzed using a one-way ANOVA with a post hoc Tukey HSD test. The data followed assumptions of the homogeneity of variance and normal distribution. To investigate the potential role of the lipophilicity and logVP inhibiting feeding behavior of *H. obscurus*, we tested correlations of the feeding behavior (weight shift) with lipophilicity and logVP (Pearson´s product–moment correlation coefficient). The analyses were performed in Statistica 7.0 software (StatSoft, Inc., Waltham, MA, USA).

## 5. Conclusions

Our study reported six isolated sesquiterpenes from essential oil and from extracts of the heartwood of *Pilgerodendron uviferum*. Additionally, we showed that two of these sesquiterpenes (−)-*trans*-calamenene (**1**) and cadalene (**2**), as well as the essential oil and DCME extract, were effective in reducing the adult growth of *Hylastinus obscurus,* a major red clover pest. The chemical composition from the heartwood of *P. uviferum* may potentially be useful in future biological control programs as a means to control the crop damage to *T. pretense* that is caused by this global pest.

## Figures and Tables

**Table 1:** Relative abundances (%) of compounds present in the essential oil (EO), petroleum ether extract (PEE), and dichloromethane extract (DCME) of *P. uviferum* heartwood. RT indicates retention time (min), RI_C_ indicates the calculated retention index, and RI_L_ indicates retention index from literature [26], MS indicates the mass spectrometry, and * indicates the relative abundances of the EO compounds (extracted from [18]).

RT	RI_C_	RI_L_	Compounds	PEE %	DCME %	EO * %	Identification
20.16	1327	1343	α-cubebene	0.09	-	0.05	RI, MS
20.82	1353	1359	copaene	0.75	0.55	0.71	RI, MS
21.13	1364	1387	β-cubebene	0.07	-	-	RI, MS
22.66	1424	1428	α-caryophyllene	0.08	0.15	1.27	RI, MS
23.13	1443	1445	bicyclosesquiphellandrene	-	0.46	-	RI, MS
23.2	1446	1462	γ-muurolene	0.33	-	-	RI, MS
23.77	1468	1469	α-muurolene	0.85	0.63	-	RI, MS
24.17	1484	1509	(−)-*trans*-calamenene	7.06	5.26	-	RI, MS,NMR
24.28	1488	1515	δ-cadinene	2.75	6.43	10.8	RI, MS
24.5	1497	1524	4,5,9,10-dehydroisolongifolene	0.27	2.14	-	RI, MS
24.6	1501	1522	α-calacorene	0.72	5.12	0.95	RI, MS
25.5	1540	1549	caryophyllene oxide	3.12	0.48	1.02	RI, MS
25.67	1547	1553	germacrene B	3.00	2.6	-	RI, MS
26.08	1565	1588	1,2-epoxyhumulene	2.06	0.34	0.51	RI, MS
26.65	1588	1620	(−)-torreyol	18.1	18.53	24.16	RI, MS,NMR
26.98	1602	1627	(−)-*epi*-cubenol	10.14	8.01	12.44	RI, MS,NMR
26.98	1602	1628	(−)-cubenol	7.0	8.1	10.5	RI, MS,NMR
27.17	1611	-	(−)-15-copaenol	14.25	12.5	15.46	MS,NMR
27.33	1618	-	cadala-1(10),3,8-triene	2.03	1.57	-	MS
27.52	1627	1652	cadalene	0.86	3.66	-	RI, MS,NMR
35.04	1991	-	7-isopropyl-1,1,4a-trimethyl-1,2,3,4,4a,9,10,10a-octahydrophenanthrene	0.19	-	-	MS
39.55	2243	-	6,7-dehydroferruginol	3.76	1.06	-	MS
39.63	2248	-	ferruginol	4.28	1.13	-	MS
42.92	2450	-	hinokione	0.26	0.25	-	MS
43.53	2489	-	hinokiol	0.29	0.75	-	MS
44.14	2529	-	sugiol	0.12	0.12	-	MS

**Table 2:** ^13^C-NMR data of sesquiterpenes isolated from *P. uviferum* (D. Don) Florin (100 MHz, CDCl_3_).

Carbon Number ^a^	^13^C Chemical Shift (ppm)					
	*trans*-Calamenene (1)	Cadalene (2)	*epi*-Cubenol (3)	Cubenol (4)	15-Copaenol (5)	Torreyol (6)
1	140.5	131.5	71.1	73.1	37.6	45.9
2	127.2	125.2	32.5	22.4	30.3	35.7
3	126.6	127.6	27.2	27.1	118.3	31.5
4	134.9	135.2	135.6	134.3	147.6	134.8
5	129.2	123.4	120.2	122.5	50.7	125.0
6	140.3	131.9	46.4	48.5	44.8	37.2
7	44.2	142.5	40.2	49.6	44.9	44.2
8	21.9	121.8	24.5	24.4	22.1	21.9
9	31.2	126.0	30.6	31.6	36.4	18.9
10	32.9	132.3	40.2	42.3	39.7	73.0
11	21.7	22.5	24.1	24.0	66.4	24.0
12	32.3	28.6	26.3	27.3	32.5	26.8
13	17.8	24.1	15.3	15.6	20.0	15.7
14	21.6	24.1	21.8	22.1	20.0	22.1
15	22.7	19.9	15.5	15.6	20.4	28.4

^a^ Assignment was performed using DEPT 135 and HSQC-ed data.

**Table 3:** ^1^H-NMR data of sesquiterpenes isolated from *P. uviferum* (D. Don) Florin (400 MHz, CDCl_3_).

Hydrogen Number ^a^	^1^H Chemical Shift (ppm)					
	*trans*-Calamenene (1)	Cadalene (2)	*epi*-Cubenol (3)	Cubenol (4)	15-Copaenol (5)	Torreyol (6)
1	-	-	-	-	2.12 m (1)	1.62 bs (1)
2	7.14 d, *J* = 8 (1)	7.95 d, *J* = 8 (1)	2.02/1.33 m (1)	1.66/1.60 m (1)	2.26 bs (2)	1.52 m (2)
3	6.97 d, *J* = 8 (1)	7.37 d, *J* = 8 (1)	2.13/1.98 m (1)	2.08 m (2)	5.48 m (1)	1.99 m (2)
4	-	-	-	-	-	-
5	7.04 s (1)	7.94 s (1)	5.41 s (1)	5.44 d, *J* = 4 (1)	1.72 d, *J* = 1 (1)	5.52 dd (1)
6	-	-	1.85 m (1)	1.67 m (1)	1.59 ^b^ m (1)	2.02 m (1)
7	2.71 m (1)	-	1.39 m (1)	1.14 m (1)	1.69 ^b^ m (1)	1.32 m (1)
8	1.84/1.61 m (1)	7.30 d, *J* = 7 (1)	1.63/1.04 m (1)	1.55/1.04 m (1)	1.53/1.59 m (1)	1.49/1.10 m (1)
9	1.98/1.34 m (1)	7.24 d, *J* = 7 (1)	1.49 m (2)	1.60/1.08 m (1)	1.73/1.63 m (1)	1.88/1.58 bs (1)
10	2.79 m (1)	-	1.35 m (1)	1.60 m (1)	-	-
11	2.31 s (3)	2.58 s (3)	1.71 s (3)	1.70 s (3)	3.98 d, *J* = 1 (2)	1.65 s (3)
12	2.26 m (1)	3.74 m (1)	2.07 m (1)	1.96 m (1)	1.51 m (1)	1.97 m (1)
13 ^b^	0.73 d, *J* = 7 (3)	1.41 d, *J* = 7 (3)	0.73 d, *J* = 7 (3)	0.80 d, *J* = 7 (3)	0.82 d, *J* = 7 (3)	0.81 d, *J* = 7 (3)
14 ^b^	1.02 d, *J* = 7 (3)		0.92 d, *J* = 7 (3)	0.87 d, *J* = 7 (3)	0.84 d, *J* = 7 (3)	0.89 d, *J* = 7 (3)
15	1.28 d, *J* = 7 (3)	2.67 s (3)	0.93 d, *J* = 7 (3)	0.96 d, *J* = 7 (3)	0.78 s (3)	1.29 s (3)

s—singlet; bs—broad singlet; d—doublet; m—multiplet; (N^o^)—integration; *J*—Hz. ^a^ Assignment was performed using COSY, HSQC-ed, and HMBC data. ^b^ Assignment could be exchanged.

## Supplementary Materials

Supplementary materials are available online.
