# Supplementary material for: Antifeedant Effects of Essential Oil, Extracts, and Isolated Sesquiterpenes from Pilgerodendron uviferum (D. Don) Florin Heartwood on Red Clover Borer Hylastinus obscurus (Coleoptera: Curculionidae)
Source: Molecules. 2018 May 27;23(6):1282. doi: 10.3390/molecules23061282 (PMC6099530; doi:10.3390/molecules23061282)
Supplement: Supplementary file 1 [file molecules-23-01282-s001.pdf]

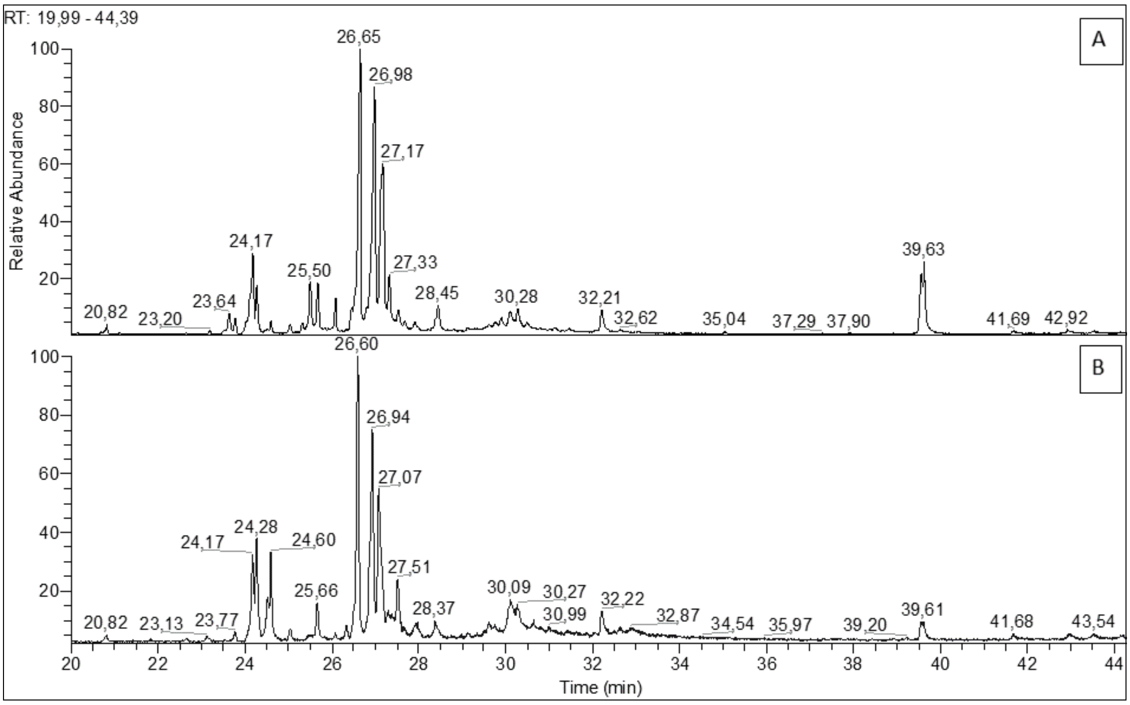

Figure S1: Chromatograms of DCME (A) and PEE (B) extracts of *P. uviferum* heartwood obtained by GC-MS analyses.
